# Public spending on orphan medicines: a review of the literature

**DOI:** 10.1186/s40545-020-00260-0

**Published:** 2020-10-13

**Authors:** Margit Gombocz, Sabine Vogler

**Affiliations:** WHO Collaborating Centre for Pharmaceutical Pricing and Reimbursement Policies, Pharmacoeconomics Department, Gesundheit Österreich GmbH (GÖG / Austrian National Public Health Institute), Vienna, Austria

**Keywords:** Orphan medicines, Pharmaceutical expenditure, Public budgets, Review, Rare diseases

## Abstract

**Background and objective:**

Little is known about how much public payers spend on orphan medicines. This study aimed at identifying information on orphan medicine expenditure incurred by public payers that was published in literature globally and at possibly synthesising their shares as portion of the total pharmaceutical expenditure.

**Methods:**

A literature review was undertaken using Medline, the Orphanet Journal of Rare Diseases and Google Scholar. Titles and abstracts were screened, and full texts of potentially qualifying studies were reviewed for inclusion. Included articles were analysed, and bibliometric parameters as well as public expenditure data on orphan medicines were retrieved.

**Results:**

Six hundred forty three articles excluding duplicates were identified. After screening of the abstracts and a review of the full texts, 13 articles qualified for in-depth analysis.

The 13 selected articles on orphan pharmaceutical expenditure were published between 2010 and 2018. Survey periods varied between 1 year and 12 years. One publication included 22 countries but the majority of the studies were related to a single country. Expenditure data was available in five of the 13 articles, and eight articles used ‘expenditure proxies’ such as sales data. Spending data had been sourced from public institutions (4 studies), private providers (5 studies) and a combination of both (3 studies, no information on data source in 1 study). In all included studies, secondary data were analysed. Reported expenditure shares for orphan medicines in relation to total pharmaceutical spend was frequently below 3%. Countries with higher shares included the USA, Canada, the Netherlands and Bulgaria—the latter reporting spending on orphan medicines as high as 9%.

**Conclusions:**

A low number of studies that informed about pharmaceutical spending on orphan medicines was published, thereof only a few explicitly analysed expenditure data of public payers. A conclusive synthesis of public spending on orphan medicines is a challenge given to the diversity in methodologies to measure expenditure. There is a need for further research to survey primary data of public spending for orphan medicines, based on a sound methodology to measure these data and to compare them internationally.

## Introduction

Rare diseases are conditions and illnesses that, per definition, affect a comparably low number of patients. The prevalence thresholds differ between countries and world regions. In the European Union (EU), for example, a rare disease is defined as a disease that affects fewer than five people in 10,000 [1]. Respective figures are fewer than 200,000 people in the United States of America (USA), fewer than 50,000 in Japan and fewer than one in 10,000 in Taiwan [2, 3].

Given low patient numbers, research and development of pharmaceuticals to treat rare diseases (so-called orphan medicines) has been promoted by governments. Thus, the EU, USA, Japan and further high-income countries offered incentives to pharmaceutical companies [4]. It has been argued that in response, manufacturers tend to focus on profitable areas such as oncology, and, as a result, orphan medicines have become non-affordable to public payers and patients as some tend to have (very) high price tags [5, 6]. Research confirmed high prices of orphan medicines [7–12] and limited access to orphan medicines, particularly in lower-resourced countries [7, 13–16]. Policymakers have to balance the objectives of access to effective medicines for the population, containment of pharmaceutical expenditure as well as long-term sustainability of the health care system and incentives for the industry. For their search for solutions, new policies to ensure access to premium-priced medicines, including orphan medicines, have been explored or are under discussion [17–19]. For instance, managed-entry agreements are frequently applied for orphan medicines [14, 20, 21]. As a standard, these agreements include a, usually confidential, discount granted to the public payer, and funding may, or may not, be linked to the health outcomes of a patient.

Concerns have been raised that a few orphan medicines may account for a comparably high share of the pharmaceutical budget of the public payers [2, 22, 23]. In addition to the high price tags, the increasing number of patients in rare diseases as well as the extension of indications are potential drivers for high shares in pharmaceutical budgets. Overall, rare diseases can be frequent, and it is assumed that 400 million people worldwide suffer from rare diseases [24]. Some studies, however, challenged the existence of a strong impact of orphan medicines on future pharmaceutical budgets [25, 26]. This debate is held against the backdrop of limited knowledge on spending for orphan medicines since expenditure for these medicines is not published as routine data in statistics in most countries.

To address this gap, the study aimed at identifying information on orphan medicine expenditure data that was globally published in literature and at possibly synthesising the shares of spending on orphan medicines as portion of the total public pharmaceutical expenditure across countries.

## Methods

A systematic literature review was undertaken in September/October 2018 using Medline, the Orphanet Journal of Rare Diseases and Google Scholar in order to identify published information about pharmaceutical expenditure on rare diseases covered by public or other third-party payers. The following search terms were applied: rare disease(s), orphan disease(s), rare condition(s), orphan condition(s), orphan drug(s), orphan medicinal product(s), rare drug(s), pharmaceutical spending, drug cost(s), expenses, pharmaceutical expenditure and budget impact.

Literature was considered eligible for further analysis if it contained information on expenditure for orphan medicines that was retrospective, not disease specific (rare diseases in total) and referred to the year 2001 and later (1 year after the Orphan Medicinal Products Regulation in the European Union had come into force). No restriction with regard to geographical scope or language was applied.

The literature selection was performed in a two-step process: first, the title and the abstract of studies were screened with regard to their compliance with the defined inclusion/exclusion criteria, and in a second step, full texts of the selected pieces of literature were studied to assess whether or not they qualified for possible inclusion.

Upon selection of eligible articles, relevant information of the included publications was summarized in an extraction matrix. The matrix contained the following information: author(s), title, year of publication, journal information, language, aim/purpose/study question, study design, country/countries of the study, methodology parameters of the expenditure data reported (sector, type of analysis, year of data, data description, data source, price level) and scope of expenditure information (e.g. on orphan medicines, public pharmaceutical expenditure, total pharmaceutical expenditure).

## Results

### Selection of the studies

Six hundred forty four articles were identified in Medline, including one duplicate. An additional search in the Orphanet Journal of Rare Diseases and a search in Google Scholar were not successful in identifying further relevant pieces of literature.

**Fig. 1:**
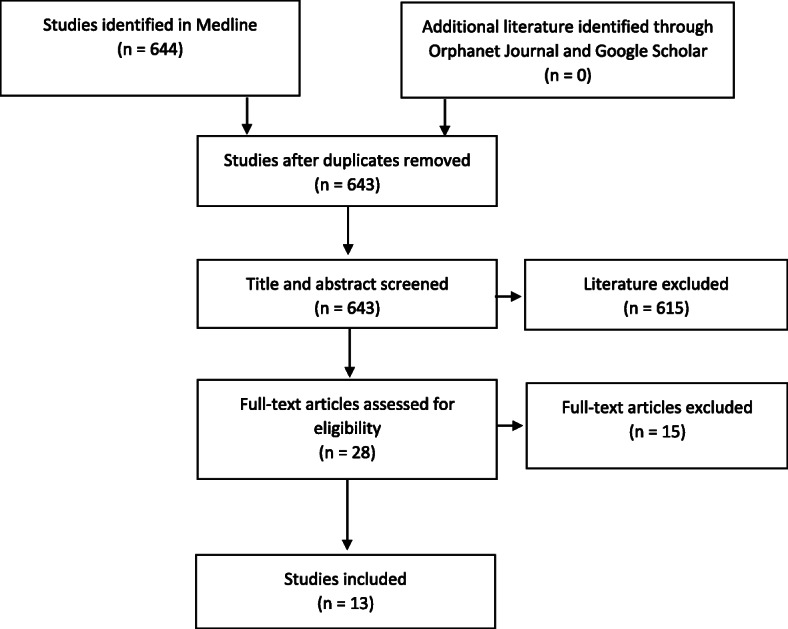
Flowchart of the literature review process

Six hundred forty three articles were screened on the basis of title and abstract, thereof full texts of 28 articles were analysed. Eventually, 13 articles qualified for the in-depth analysis (see the literature review process in Fig. 1).

### Characteristics of included studies

The 13 included articles were published between 2010 and 2018, thereof three in 2018, three in 2016, one in 2015, three in 2014, one in 2011 and two in 2010. In some cases, investigated data in these studies were related to earlier periods of time, such as the years 2006 and 2007 and even 2003 in one study. Survey periods varied between 1 year and 12 years. The number of countries analysed ranged from 1 to 22; however, most of the studies were related to a single country.

Spending data were mainly reported from countries in the EU and also from Canada, Taiwan, Turkey and the USA. The majority of studies (8) investigated retrospective spending data on orphan medicines, whereas the remaining (5) reported both retrospective and prospective orphan medicine expenditure. In five of the 13 studies, expenditure data were actually available, compared with eight studies with ‘expenditure proxies’: six studies were based on sales data, and two studies provided some type of cost data. In four studies, information sourced from a public data provider was analysed, while data were obtained from private providers in five studies and from a combination of public and private data providers in three studies (in one study, the data source was not traceable). Pharmaceutical expenditure data were indicated for both inpatient and outpatient sectors in three articles; in nine studies, the sectors were not defined. None of the studies was based on a primary data collection; thus, solely secondary data were analysed. In all but one study, national annual spending data were expressed either in absolute terms or as a share of total public pharmaceutical expenditure (frequently called ‘total pharmaceutical expenditure’ even if they were related on the public sector only), or both; one study provided a cumulative percentage rate over all surveyed countries.

### Synthesis of included studies’ results

Taiwan experienced a nine-fold increase in spending on orphan medicines in absolute terms in a period of 12 years, and the Netherlands a 3.3-fold growth in the outpatient sector and a 4.2-fold increase in the inpatient sector during 7 years. Public spending on orphan medicines more than doubled in Bulgaria from 2011 till 2014. In comparison, the growth rates for Latvia (a 20% increase from 2010 to 2014) seemed to be rather low.

In terms of total public expenditure, reported public spending on orphan medicines ranged from less than 1% in Taiwan in 2003 to more than 9% in Bulgaria in 2014.

The authors of the analysed studies reported different methodologies and limitations of their research, including short observation periods, possible over- or underestimation due to methodological issues and uncertainties were related to the projections due to calculations based on historical data and the scope of medicines in the studies (Table 1).

**Table 1:** Public spending on orphan medicines reported in included literature

Authors and year of publication	Aim of the study	Type of survey and expenditure data sources	Included country/countries	Year of data	Spending data	Sector	OM spending		Limitations reported in the studies
							In absolute terms	As percentage	
Kanters TA, Steenhoek A, Hakkaart L (2014) [23]	To assess uptake and budget impact of OM	Secondary data analysis of GIP database (OP) and information by ph. c. or Monitor Expensive Drugs and FarmInform (IP)	NL	2006–2012	Yes (OP) and no (IP), BI (predicttions)	OP and IP	2006 OP: 52.7 mill. EUR IP: 61.2 mill. EUR 2007 OP: 68.7 mill. EUR IP: 97.9 mill. EUR 2008 OP: 97.8 mill. EUR IP: 158.6 mill. EUR 2009 OP: 118.1 mill. EUR IP: 192.7 mill. EUR 2010 OP: 141.6 mill. EUR IP: 225.9 mill. EUR 2011 OP: 156.2 mill. EUR IP: 241.4 mill. EUR 2012 OP: 175.2 mill. EUR IP: 260.4 mill. EUR	2006: 1.1% of TPE 2007: 1.6% of TPE 2008: 2.6% of TPE 2009: 3.0% of TPE 2010: 3.6% of TPE 2011: 3.8% of TPE 2012: 4.2% of TPE	- Limited observation period (7 years)
Orofino J, Soto J, Casado MA, Oyagüez I (2010) [27]	- To describe the status of orphan medicines in 2007 in the five countries in the EU with the greatest pharmaceutical expenditure - To estimate the mean annual cost per patient and indication in relation to orphan medicines - To determine the percentage contribution of orphan medicines to overall spending on medicines in each of these five countries in 2007	Secondary data analysis of IMS Health, MIDAS database	DE, ES, FR, IT, UK	2007	No, sales data	n.a.	-	2007 FR: 1.7% of overall pharmaceutical expenditure DE: 2.1% of overall pharmaceutical expenditure IT: 1.5% of overall pharmaceutical expenditure ES: 2.0% of overall pharmaceutical expenditure UK: 1.0% of overall pharmaceutical expenditure	- Pharmaceutical costs only had been considered (no direct or indirect treatment costs) - Calculations based on regimen in SPC - Short assessment period of pharmaceutical expenditure (1 year) - Prevalence data not completely trustworthy - Standardised information provided by IMS Health, MIDAS database (data collection method used in each country could be biased)
Hutchings A, Schey C, Dutton R, Achana F, Antonov K (2014) [28]	- To examine historical trends in OM designation, market authorization, sales and budget impact from 2000 to 2012 - To predict the evolution in OM use for existing diseases and new indications between 2013 and 2020	Secondary data analysis of GERS (France) and IMS Health, MIDAS database (France)	FR, SE	2004 2006 2012^a^	No, sales data	n.a.	-	FR: 2012: 3.1% of TPS SE: 2006: 0.7% of TPS 2012: 2.5% of total pharm. market value	- Forecasting assumptions
Schey C, Milanova T, Hutchings A (2011) [25]	To estimate the European budget impact of orphan medicines as a percentage of total pharmaceutical expenditure, between 2010 and 2020, based upon 10 years of orphan medicine experience in Europe.	Secondary data analysis, data source not indicated	AT, BE, CY, DE, EE, ES, FI, FR, EL, IE, IT, LU, MT, NL, PT, SK, SI, UK	2010	n.a.	n.a.	-	2010: Cumulative for all countries 3.3% of total pharmaceutical spending	- Orphan disease rather than the individual orphan medicine used for modelling - Prevalence data might be weak due to data source - Used ex-factory prices may not reflect effective price paid - Predictability of prices after patent expiry - Pharmaceutical market growth rate, success rate and uptake rate may be uncertain
Denis A, Mergaert L, Fostier C, Cleemput I, Simoens S (2010) [22]	- To calculate the impact of OM for 2008 - To forecast its impact over the following 5 years	Secondary data analysis of data in ministerial decrees, via NIHDI, Ministry of Economic Affairs, IMS Health	BE	2008	Expenses estimated based on treatment costs	n.a.	2008: 66.2 mill. EUR	2008: 1.9% of TPE	- One product excluded due to missing information - Pharmaceutical expenditure only (no total treatment costs considered) - Products financed by a special fund not considered - Possible lower prices in future not considered
Iskrov G, Jessop E, Miteva-Katrandzhieva T, Stefanov R. (2015) [29]	To estimate the impact of OM on NHIF total pharmaceutical budget between 2011 and 2014	Secondary data analysis of NHIF	BG	2011 2014^b^	Yes	n.a.	2011: 31.6 mill. BGN 2014: 74.5 mill. BGN	2011: 6.0% of TPE 2014: 7.8% of TPE	None reported
Iskrov GG, Jakovljevic MM, Stefanov SS (2018) [30]	To estimate the budgetary impact of rare disease medicines’ therapies from NHIF perspective for 2014 and 2016 - To compare the main cost drivers for this period	Secondary data analysis of NHIF	BG	2014 2016	Yes	OP and IP^c^	-	2014: 9.39% of TPE 2016: 9.25% of TPE	- Included both orphan and non-orphan medicines (rare disease indications used for analysis) - Analysis with official list prices, therefore BI might be overestimated
Logviss K, Krievins D, Purvina S (2016) [31]	To assess the budget impact of OM in Latvia and compare it with other European countries	Secondary data analysis of NHS	LV	2010–2014	Yes	n.a.	2010: 2.1 mill. EUR 2011: 2.6 mill. EUR 2012: 3.1 mill. EUR 2013: 2.1 mill. EUR 2014: 2.6 mill. EUR	2010: 1.95% of TPM 2011: 2.16% of TPM 2012: 2.62% of TPM 2013: 1.83% of TPM 2014: 2.16% of TPM	- Payers’ expenditure perspective only - Product costs exceeding a yearly limit of NHS are not considered (costs might be higher) - Different approach for estimating the number of patients
Divino V, DeKoven M, Kleinrock M, Wade RL, Kaura S (2016) [32]	To estimate the economic impact of OM in the period 2007–2013 - To extrapolate orphan medicine spending up to 2018	Secondary data analysis of IMS Health, MIDAS database	US	2007–2013	No, sales data	n.a.	2007: 15.0 bill. USD 2008: 17.1 bill. USD 2009: 19.4 bill. USD 2010: 23.1 bill. USD 2011: 26.1 bill. USD 2012: 28.0 bill. USD 2013: 30.0 bill. USD	2007: 4.8% of TPS 2008: 5.5% of TPS 2009: 6.0% of TPS 2010: 6.8% of TPS 2011: 7.5% of TPS 2012: 8.5% of TPS 2013: 8.9% of TPS	- No stratification between therapies for chronic and acute illnesses (potential long-term impact on payers’ expenditure) - IMS Health, MIDAS database do not cover 100% of the market - No generic orphan medicines considered - Potential off-label use of orphan medicines not considered
Divino V, DeKoven M, Kleinrock M, Wade RL, Kim T, Kaura S (2016) [33]	- To estimate the financial impact of OM on the TPE from 2007 to 2013 in Canada - To extrapolate orphan medicine spend up to 2018	Secondary data analysis of IMS Health, MIDAS database	CA	2007–2013	No, sales data	OP and IP	2007: 610.2 mill. CAD f2008: 669.2 mill. CAD 2009: 743.7 mill. CAD 2010: 818.1 mill. CAD 2011: 880.5 mill. CAD 2012: 989.6 mill. CAD 2013: 1,100.0 mill. CAD	2007: 3.3% of TPS 2008: 3.4% of TPS 2009: 3.6% of TPS 2010: 4.0% of TPS 2011: 4.4% of TPS 2012: 5.0% of TPS 2013: 5.6% of TPS	- IMS Health, MIDAS database does not cover 100 % of the market - Custom methodologies - Possible changes through policy adoption not considered - Potential differences in indication approvals (no approval in the USA, not accounted for in the study) - No generic orphan medicines considered - Potential off-label use not considered
Kockaya G, Wertheimer AI, Kilic P, Tanyeri P, Vural IM, Akbulat A, Artiran G, Kerman S (2014) [34]	To shed light on the use of OM in Turkey to aid further classifications of rare diseases and assessments of orphan medicines in the country	Secondary data analysis of IMS Turkey and TITCK	TR	2008–2010	No, sales data	n.a.	2008: 135.7 mill. EUR 2009: 182.4 mill. EUR 2010: 208.5 mill. EUR	2008: 2% of TPE 2010: 3% of TPE	None reported
Hsu JC, Wu H-C, Feng W-C, Chou C-H, Lai EC-C, Lu CY (2018) [2]	To examine 2003–2014 longitudinal trends in the prevalence and expenditure of rare disease s in Taiwan	Secondary data analysis of NHIRD	TW	2003–2014	Yes	n.a.	2003: 13.2 mill. USD 2004: 17.7 mill. USD 2005: 21.5 mill. USD 2006: 30.8 mill. USD 2007: 41.3 mill. USD 2008: 49.2 mill. USD 2009: 54.6 mill. USD 2010: 61.8 mill. USD 2011: 72.7 mill. USD 2012: 91.5 mill. USD 2013: 104.9 mill. USD 2014: 122.0 mill. USD	2003: 0.35% of TPE 2004: 0.41% of TPE 2005: 0.50% of TPE 2006: 0.73% of TPE 2007: 0.99% of TPE 2008: 1.14% of TPE 2009: 1.21% of TPE 2010: 1.37% of TPE 2011: 1.51% of TPE 2012: 1.92% of TPE 2013: 2.06% of TPE 2014: 2.31% of TPE	- Nationwide approach instead of individual patients (no out-of-pocket payments or clinical outcomes considered) - Focus on rare diseases in general (no analysis with regard to certain rare diseases except for 2 rare diseases)
Deticek A, Locatelli I, Kos M (2018) [35]	To estimate patient access to different medicines for rare diseases from the comprehensive Orphanet list in various European countries in the past decade	Secondary data analysis of IMS Health data	AT, BE, BG, CH, CZ, DE, EL, ES, FI, FR, HR, HU, IE, IT, NL, NO, PL, RO, SE, SK, SI, UK	2014^d^	No, sales data	OP and IP^e^	AT: 4 mill. EUR/inh. BE: 11 mill. EUR/inh. BG: 4 mill. EUR/inh. CH: 12 mill. EUR/inh. CZ: 2 mill. EUR/inh. DE: 15 mill. EUR/inh. EL: 0.2 mill. EUR/inh. ES: 8 mill. EUR/inh. FI: 7 mill. EUR/inh. FR: 12 mill. EUR/inh. HR: 3 mill. EUR/inh. HU: 2 mill. EUR/inh. IE: 7 mill. EUR/inh. IT: 12 mill. EUR/inh. NL: 7 mill. EUR/inh. NO: 6 mill. EUR/inh. PL: 1 mill. EUR/inh. RO: 2 mill. EUR/inh. SE: 9 mill. EUR/inh. SI: 8 mill. EUR/inh. SK: 6 mill. EUR/inh. UK: 11 mill. EUR/inh.	-	- IMS Health data might not reflect the actual access to orphan medicines in the studied countries - Expenditures might be overestimated (products with more than one indication that are not for rare diseases) - Sales data only included if sales was continuous over a certain time - Number of patients in need of treatment might differ from country to country due to prevalence of diseases and potential prescribing restrictions

Countries: *AT* Austria, *BE* Belgium, *BG* Bulgaria, *CH* Switzerland, *CY* Cyprus, *DE* Germany, *EE* Estonia, *EL* Greece, *ES* Spain, *FI* Finland, *FR* France, *HR* Croatia, *HU* Hungary, *IE* Ireland, *IT* Italy, *LU* Luxembourg, *LV* Latvia, *MT* Malta, *NL* Netherlands, *NO* Norway, *PL* Poland, *PT* Portugal, *RO* Romania, *SK* Slovakia, *SI* Slovenia, *TR* Turkey, *TW* Taiwan, *UK* United Kingdom, *US*A United States of America

Currencies: *BGN* Bulgarian Lev, *CAD* Canadian dollars, *EUR* Euro, *USD* US dollars

Other abbreviations: *BI* budget impact, *bill.* billion, *GERS* Groupement pour l’Elaboration et la Réalisation de Statistiques, France, *GIP* Drug Information Project database by Health Care Insurance Board, Netherlands, *inh*. inhabitant, *IP* inpatient, *mill.* million, *NHIF* National Health Insurance Fund, *NHIRD* National Health Insurance Research Database, *NHS* National Health Service, *NIHDI* National Institute for Health and Disability Insurance, *n.a.* not available, *OM* orphan medicine, *OP* outpatient, *ph. c.* pharmaceutical company, *SPC* summary of product characteristics, *TITCK* Turkish Medicines and Medical Device Agency—Türkiye I˙laç ve Tıbbi Cihaz Kurumu, *TPE* total pharmaceutical expenditure, *TPM* total pharmaceutical market, *TPS* total pharmaceutical sales

^a^ Data were observed in the period 2000–2012. Figures are solely available for the years 2004, 2006 and 2012

^b^ Data were observed in the period 2011–2014. Figures are solely available for the years 2011 and 2014

^c^ Inpatient data refer solely to oncology treatments

^d^ Data were observed in the period 2005–2014. Figure is solely available for the year 2014

^e^ The treatment sector related to sales data varied between countries

## Discussion

The study reviewed existing evidence in literature on the availability of public spending data on orphan medicines, with a view to understanding the relevance of pharmaceutical spending for these medicines.

Overall, the number of studies that were eligible for inclusion in the analysis was rather limited, but some spending data on orphan medicines were published in literature in the last decade. Improved availability of such information might be attributable to an increased interest in these medicines, given the market entry of several high-priced medicines in recent years and concerns of policymakers [36–39]. Also, apart from the USA that introduced legislation for orphan medicines 35 years ago, orphan medicine legislation in other countries and regions was mostly introduced in the last decades (e.g. Singapore 1991 [40], Japan in 1993 [4], Australia in 1997 [41], the EU in 2000 [1], Taiwan 2000 [42]). Legislation for orphan medicines has mainly been implemented in high-income countries [4], and data on spending for orphan medicines were predominantly reported for these countries. This may also be linked to other reasons such as publication and manuscript selection bias with regard to lower-income countries [43–45], advanced statistical data and frameworks available in higher-income countries (e.g. the System of Health Accounts methodology [46] developed by the Organisation for Economic Co-operation and Development) and availability of a higher number of medicines that have been defined as orphan medicines in higher-income countries [15, 47, 48].

In the light of current developments (i.e. a growing number of orphan medicines being authorised and also marketed, e.g. [49]), it could be expected that spending on orphan medicines would have increased over the years. In fact, almost all included studies that provided data for a longer period of time confirmed a growth in spending on orphan medicines.

There is variation in the shares of pharmaceutical spending on orphan medicines between the analysed countries. For the year 2013, studies reported shares of public spending on medicines that ranged from 1.83% in Latvia [31] and 2.06% in Taiwan [2] to 5.6% in Canada [33] and 8.9% in the USA [32]. Two studies reported different findings on 2014 data for Bulgaria (7.8% [29] and 9.39% [30]) even if the data had been retrieved from the same source. A study published after the authors had finalised their search showed similar figures for eight European countries: spending shares on orphan medicines as a portion of total pharmaceutical expenditure ranged from 1.95 to 6.18% in 2013 and from 2.5 to 6.84% in 2014 [13]. While the majority of the articles included in this review reported shares of orphan medicine expenditure of around 2–3%, some studies identified shares that exceeded 5% and even 7–8%. Such figures could be considered challenging and even alarming, given the tendency of growth in pharmaceutical expenditure for these medicines. Even if parts of the variations might be attributable to the methodological design of the analysed studies, a significant and rising share of pharmaceutical expenditure for comparatively low consumption urges policymakers to develop appropriate action to balance the aims to ensuring access to medicines and guaranteeing long-term sustainability of the health care system. In any case, the arguments made by some authors that orphan medicines would not have a strong impact on future pharmaceutical budgets [21, 22] cannot be confirmed by the data retrieved in this literature review.

The review identified methodological limitations in the existing evidence of public spending on orphan medicines published in literature. This also limits cross-country comparability of the data.

First, different national definitions of orphan medicines were applied, so the scope of medicines included as orphan medicines into national data varied between countries.

Second, some studies [32, 33, 35] included all medicines with a designation as an orphan medicine, independent from actual validity of the orphan status, whereas other research [31] considered solely orphan medicines with a valid orphan status (e.g. awarded by the European Medicines Agency in the EU). Apart from one study [25], it was not clear for the articles relating to EU Member States whether or not medicines for treating rare diseases before the introduction of the Orphan Medicinal Products Regulation in 2000 had been included.

Third, comparability was impaired by the reporting of national spending data for different price types (e.g. ex-factory price, consumer price), and some articles did not even provide any information on the price type at which spending data was measured.

Fourth, not all studies reported spending data but some considered sales data as kind of proxy for pharmaceutical spending.

Fifth, some studies did not provide any information if their data were related to public or private expenditure. In general, given the character of these medicines (high prices, specific application that may require inpatient use) and the data mainly sourced from solidarity-based high-income countries, public funding of orphan medicines is highly likely. This assumption is also supported by the fact that some studies (e.g. on EU Member States) surveyed and analysed data from sources managed by public payers.

### Limitations

The study has some limitations. It focused on articles in peer-reviewed journals because there was interest to see what has been published. It is, however, acknowledged that further studies and statistics of primary data might be available publicly (e.g. on a website), but they are not published in peer-reviewed literature. Furthermore, the study investigated spending data (including proxies for spending) but did not consider information on contributing factors for expenditure, such as the number of authorised or marketed orphan medicines (reported in some articles [22, 23, 27, 32–35]) and the number of treated patients [22, 23, 29]. Finally, the literature search was performed in September/October 2018, and the authors are aware that after completion of the literature review, at least one article on orphan medicines and spending [13] was published.

### Call for further action

The review identified a range of shares related to public spending for orphan medicines from a rather limited body of literature. The relevance of the findings is challenged by the diversity of the definitions and measurement methodologies applied to the underlying data. Further research is needed. As a first step, it is suggested to complement this literature review by a search of primary public expenditure data on orphan medicines in national statistics. In this context, it is advised to analyse national definitions of these spending data and to explore cross-country comparability. Furthermore, to bridge gaps in cases of non-reporting in publicly accessible sources, it is recommended to address experts in public authorities (such as medicine agencies and public payers’ institutions) to provide these data, ideally in accordance with definitions pre-specified by researchers, in order to allow for follow-up international comparisons. Researchers are advised to pilot these surveys in countries with good statistics and then roll out globally. It should be aimed for the inclusion of low- and middle-income countries in these studies, though researchers should also take into account that the statistical basis for pharmaceutical expenditure data in general might need to be installed first in some countries. The methodology development could benefit from cross-country collaboration, with a possible involvement of international institutions.

The study findings do not only call for further research but also for action of policymakers. While research can offer exploratory work to generate evidence and can contribute to methodology development, also with a view to ensuring international comparability, it would be subsequently up to the public authorities to decide on a framework that provides for regular survey, analysis and publication of public spending data for orphan medicines. This would allow establishing an internationally comparable system of orphan medicines spending account that could, in the longer run, be implemented globally, including in middle- and low-income countries.

## Conclusions

The study stressed the limited body of evidence on public spending for orphan medicines, as only few studies had been published, and some research used proxy data such as sales figures to inform on public spending. Some single-country studies pointed to a steady increase in the spending portion on orphan medicines over the years. While some studies reported a share of around 2–3% of pharmaceutical spend on these medicines, there was considerable variation in the figures (0.35%, Taiwan, 2003 and 9.39%, Bulgaria, 2014).

Despite methodological limitations in the retrieved literature which may be one explanatory factor for the diversity in outcome data, the comparatively high shares on spending for orphan medicines in some countries and their increases are key findings of policy relevance. They call for further monitoring of orphan medicine expenditure, analysing their causes and taking policy action if required.

As a prerequisite, the evidence base would need to be established in numerous countries. Overall, this review highlights the need for generating further information. More research to survey national primary spending data is required. Policymakers and researchers are called upon to collaborate on developing a robust methodology to survey and publishing public spending on orphan medicines as routine data.

## Data Availability

All data generated or analysed during this study are included in this published article.

## Abbreviations

EU: European Union
USA: United States of America
